# Indoor Air Quality in an Orthopedic Hospital from Romania

**DOI:** 10.3390/toxics12110815

**Published:** 2024-11-13

**Authors:** Flaviu Moldovan, Liviu Moldovan

**Affiliations:** 1 Orthopedics—Traumatology Department, Faculty of Medicine, George Emil Palade University of Medicine, Pharmacy, Science, and Technology of Targu Mures, 540142 Targu Mures, Romania; 2 Faculty of Engineering and Information Technology, George Emil Palade University of Medicine, Pharmacy, Science, and Technology of Targu Mures, 540142 Targu Mures, Romania; liviu.moldovan@umfst.ro

**Keywords:** hospital, orthopedic, carbon dioxide, nitrogen dioxide, particulate matter, volatile organic compounds

## Abstract

Inside hospitals, there is a trend of increasing levels of air pollutants. However, only the indoor air quality in operating theaters is carefully monitored. Therefore, we set the goal of this study to evaluate the indoor air quality in areas of an orthopedics department and to compare the indoor air quality indices that characterize these areas. We used a monitoring system based on the Internet of Things with uRADMonitor model A3 sensors, with which we prospectively measured indoor air quality in the facilities of the orthopedic emergency hospital of Targu Mures in Romania, between 1 February 2023, and 31 January 2024. The primary target pollutants investigated in the emergency room, outpatient room and ward were carbon dioxide (CO_2_), nitrogen dioxide (NO_2_), volatile organic compounds (VOCs) and particles with a diameter smaller than 2.5 μm (PM2.5). We compared the effectiveness of the intervention for emergency rooms where air purifiers were working or not. The concentrations of CO_2_, VOCs and PM2.5 were significantly higher in the emergency room than in the outpatient room or ward. The indoor air quality was worst in winter, when the CO_2_, NO_2_ and VOC concentrations were at their highest. Air purifiers can help reduce the concentration of PM2.5 in emergency rooms. Medical staff and patients in orthopedic hospitals, especially in emergency rooms, are frequently exposed to polluted ambient air, which can affect their health. Orthopedic medical practice guidelines should address issues relating to the protection of personnel through the application of measures to improve indoor air quality.

## 1. Introduction

Since people typically spend more than 90% of their time indoors, a fundamental element that affects their well-being and health is indoor air quality. In order to improve the health, comfort and well-being of building occupants, environmental governments, political institutions and the international scientific community have been placing increasing importance on indoor air quality in recent decades [1], and the topic is intensively researched [2,3]. The direct or indirect effects of damaged air quality affect the health of building occupants, which can cause annual deaths of over half a million in Europe [4]. For these reasons, the monitoring of indoor air quality is a relevant issue that requires continuous concern to ensure occupational health through improved living environments in the workplace [5,6,7]. To reduce health risks, effective policies and interventions need to be implemented that can be supported by early identification and adequate research [8].

The quality of indoor air and ventilation of buildings has been assessed since several decades ago by the concentration of carbon dioxide (CO_2_). This is also an indicator of the risk of contracting respiratory infections [9]. Values above 1000 ppm indicate potential indoor air problems [10]. Also, the difference between indoor and outdoor carbon dioxide concentrations should be considered. Indoor CO_2_ levels should not exceed the local outside air concentration by more than 650 ppm, as recommended by ASHRAE [11]. CO_2_ generation rates can be determined from concepts related to human metabolism, body size and composition and the intensity of the physical activities of building occupants [12]. Through their real-time assessment, buildings can be quickly intervened in to eliminate problems. It is also possible to anticipate the need for technical interventions that can ensure a healthier living environment.

The deterioration of indoor air quality is due to pollutants such as particulate matter (PM), volatile organic compounds (VOCs), formaldehyde (HCHO), carbon monoxide (CO), sulfur dioxide (SO_2_), polycyclic aromatic hydrocarbons (PAH), nitrous oxide (NO), pollen, microbial spores, allergens, etc. Some of these are due to indoor sources such as carpets, paints and building materials [13]. A wide variety of resuspension activities increase human exposure to particulate matter. PM2.5, PM5 and PM10 contribute to the “personal cloud” effect. These are due to indoor activities that disturb dust reservoirs on furniture and textiles, such as folding clothes and blankets, making a bed, dry dusting, etc. The type of flooring in the premises and the intensity of activities are also important factors for dust resuspension [14]. The location of the hospital can also influence the indoor air quality and concentrations of PM2.5, submicron particles, heavy metals and microbial contaminants; the highest of these concentrations are registered in the case of hospitals located in industrial areas [15].

Due to the increase in outdoor pollution, the need to develop technologies to clean the air inside buildings has also appeared. Effective ventilation is necessary to reduce contamination inside sanitary facilities due to CO_2_, NO_2_ and O_3_ concentrations but also affects their relationship with other indoor environmental factors [16]. Along with traditional systems that control PM2.5/10, there is a need to develop more advanced control systems that sustainably reduce PM levels. Among them is the use of electromagnetic waves in the control of air pollution [17], the discovery of new materials for sensors, and also the construction of smart buildings, with the support of which promising strategies can be developed for the control and improvement of indoor air quality [18].

The rapid increase in the level of air pollution generates adverse health effects, such as allergic diseases [19]. Indoor air pollution also plays an important role in the development of lung cancer [20]. Volatile organic compounds (VOCs) have adverse effects on lung function by increasing oxidative stress [21]. Significant positive associations with ambient concentrations of PM2.5, PM10, CO, SO_2_ and NO_2_ are reported for cardiovascular disease and respiratory disease [22]. Higher exposures to NO_2_, PM2.5 and SO_2_ have been associated with a higher risk of cardiovascular disease [23]. Chronic exposure to ambient air pollution appears to increase the level of pro-inflammatory markers in the human brain, which can lead to neuroinflammation, neurodegeneration and brain barrier failure [24]. Air pollutants are also associated with an increased risk of outpatient visits [25].

Usually in hospitals, the indoor air quality is monitored in radiology departments, intensive care units and operating rooms, without monitoring in wards with beds, outpatient rooms and treatment rooms [26]. Although there are mechanical ventilation systems for the air inside hospitals, high levels of carbon monoxide, bioaerosols and chemical compounds persist. Hospitals have indoor environments that make up complex ecosystems and that, for improvement, require a study of factors with the potential for modification [3]. This requires surveillance systems for all hospital wards, which must be properly designed, built and operated, along with staff training. In this way, public healthcan be kept under control by limiting the airborne transmission of infectious respiratory diseases in an environment where indoor air quality is substantially affected [27]. In wards where staff face a high cognitive load, indoor air quality assessment using CO_2_ concentration can be facilitated by implanting detection network systems based on the Internet of Things (IoT) [28,29]. This system requires the installation of sensors, data collection devices and network infrastructure that combines applications of grid computing and cloud technology through which a real-time indoor air quality monitoring network is created that is efficient and operates at low costs. The World Health Organization (WHO) issued air quality guidelines and estimated reference levels for pollutants [30,31]. WHO guides healthcare facilities on indoor environmental quality assessment with regards to the identification of pollutants, control, methods for the analysis and measurement of indoor environmental parameters and maintenance to ensure a healthy environment [32].

Doctors and medical staff from the specialty of Orthopedics–Traumatology spend most of their time in the orthopedic hospital alongside patients in the outpatient room, emergency room, ward or operating room. These spaces are closed and exposed to gasses expelled by people in the hospital when using disinfectants but also during various procedures such as incision, drainage, biopsy, electrocautery, punctures and intra-articular infiltrations. Casting immobilization maneuvers, instrument cleaning and disinfection can increase air pollution [33]. The orthopedics department uses many medical supplies without their life cycle assessment quantifying emissions over their lifetime. Better informed choices of methods and materials used in orthopedic care would allow for reducing pollution and their carbon footprint [34], as is performed in the medical specialties of dentistry [35,36] or private healthcare and elderly care facilities [37], which may have a higher pollution potential than in orthopedics. Air control measures are essential to reduce the spread of biological particles [38]. The evaluation of the effectiveness of air control is also necessary to detect the irregular introduction of particles into the air through the clothing of medical staff and visitors as well as through the transport of medical and personal materials in all hospital departments [39]. From this analysis, it can be noted that orthopedic hospitals differ from general hospitals or other types of specialized hospitals by the type of specific interventions that are performed and that generate specific pollution.

Along with indoor spaces, there are various other areas that remain crucial in assessing levels of air pollution where people are forced to breathe poor quality air due to excessive sources of pollution. This is the case for indoor university campuses equipped with personal computers [40]; underground garages; covered parking lots and other similar structures, where a person entering the area is forced to inhale airborne pollutants [41]; other outdoor environments for urban walking routes on foot through areas with different traffic densities; and people waiting at a bus stop or standing next to a traffic light or along a busy road, as currently happens in large cities [42].

With the support of controversies revealed by the specialized literature regarding the high risks related to the poor indoor air quality inside the hospital, we formulated the following research question:

(RQ) Are there differences in indoor air quality between different areas of the orthopedic hospital that are perceived by medical staff and can cause health problems for them?

The aim of this study was to assess the indoor air quality in the areas of the orthopedic hospital and to compare indoor air quality indices to formulate improvement measures.

## 2. Materials and Methods

### 2.1. The Research Design

This quantitative, exploratory, primary research was designed to investigate the research question posed in the introductory section. For this, we used a monitoring system based on the Internet of Things with uRADMonitor model A3 sensors [43] to prospectively measure indoor air quality indices in an orthopedic hospital. The uRADMonitor model A3 sensors measure air temperature (in the interval −40 °–+85 °C), barometric pressure (300 hPa–1100 hPa), humidity (0% RH–100% RH) and volatile organic compounds (0 mg/m^3^–100 mg/m^3^ reducers, 10 mg/m^3^ oxidizers) using Bosch’s BME680. It detects the concentration of PM2.5 particles in the air (0 μg/m^3^–1000 μg/m^3^) using high-quality laser scattering sensors. The measurement of CO_2_ concentration in the air (400 ppm–5000 ppm) was performed by an electrochemical formaldehyde sensor and a non-dispersive infrared sensor. Gamma ionizing radiation and X-rays (0.01 μSv/h–9999.99 μSv/h) were detected by a SI29BG Geiger tube. The detection elements are subjected to an active air flow generated by a fan incorporated in the sensor. The primary target pollutants investigated were carbon dioxide (CO_2_), nitrogen dioxide (NO_2_), volatile organic compounds (VOCs) and particles with a diameter smaller than 2.5 μm (PM2.5). The measurements were carried out over a period of one year, between 1 February 2023 and 31 January 2024. Recordings were made by continuous surveillance and a permanent data flux. The hospital building has a centralized heating system that uses natural gas as fuel, and the thermal agent is circulated in the rooms through copper pipes and aluminum radiators. In order not to alter the results of the study, we did not allow the use of heaters, fans, coolers or additional personal cleaning devices [44].

Following the research question formulated in our study, we investigated the differences in indoor air quality recorded in the areas of the Orthopedics–Traumatology department at the County Emergency Clinical Hospital of Targu Mures. To compare the air quality, we mounted sensors and performed measurements in three areas of the orthopedic hospital: the emergency room, outpatient room and ward. These premises are located on distinct levels: the emergency room is located on the ground floor, the outpatient room is located on level 1 and the ward is located on level 2.

**Figure 1:**
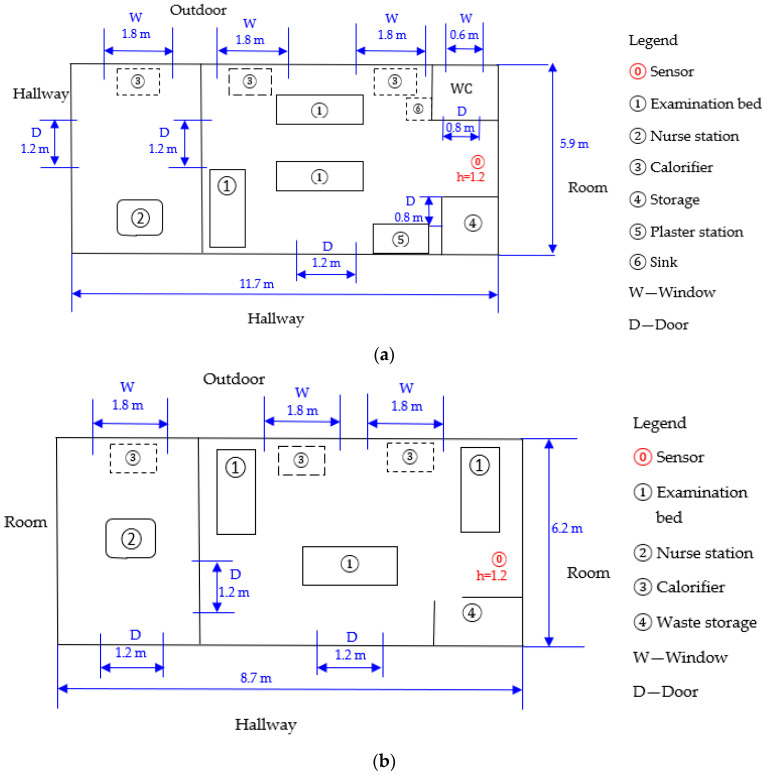

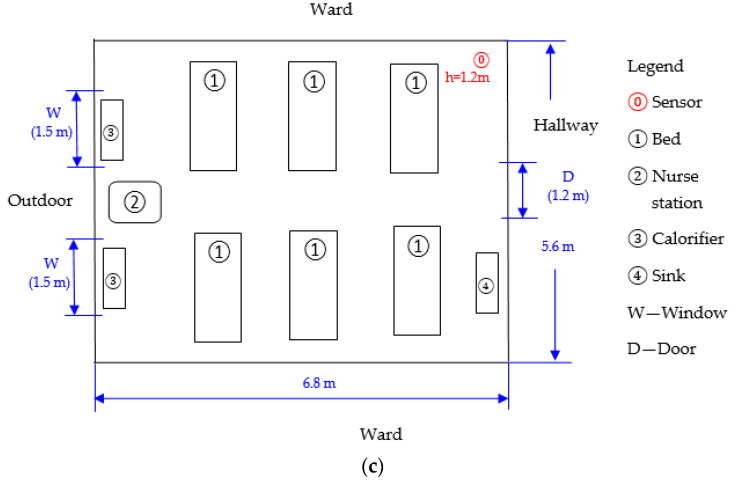
Plans of the investigated areas: (**a**) emergency room; (**b**) outpatient room; (**c**) ward.

The scheme of the measurement plans of the investigated areas, with measurement points and heights above the ground, are presented in Figure 1.

The ventilation and air conditioning of the entire hospital is carried out with the support of air handling units which are connected to an energy management and monitoring system. The microclimate parameters in the three areas were in intervals that did not affect the values of the pollutant parameters: ambient pressure 98–101 kilopascals [kPa], temperature 18–24 °C, relative humidity 35–45% and airflow velocity less than 0.25 [m/s]. During the investigated period, the concentration of CO_2_ in the atmosphere outside the hospital was approximately 350 ppm.

The records related to consultations, medical interventions and hospitalizations allowed us to establish that the outpatient room is visited daily by approximately 59 patients; in the emergency room, an average of 72 interventions are performed every day, and the ward has an average of 6 hospitalized patients in a room. The sources of pollution are due to the main operations carried out in the hospital departments which are extremely diverse. In general, in the outpatient room, consultations are held by appointment, intra-articular infiltrations are performed, sterile dressings are changed and damaged immobilizations are restored. In the emergency room, the medical staff perform emergency consultations, local anesthesia maneuvers, fracture reduction procedures and immobilization in plaster casts/special bandages and intra-articular punctures. In the ward, various types of procedures are performed, which include cleaning wounds with antiseptic solutions and sterile dressings, carrying out continuous tarsal skeletal traction and immobilization on Bohler–Braun type splints under local anesthesia, medication administration and physiotherapy programs with dedicated devices.

We assumed that the patient reception areas of the emergency room and outpatient room are representative of the high risk of indoor air pollution, while the ward presents a lower risk of pollution.

We studied the seasonal variations in the indoor air quality by grouping and classifying the months into the four seasons: spring (the months of March–May), summer (the months of June–August), autumn (the months of September–November) and winter (the months of December–February).

Given that the emergency rooms are the most polluted areas of the orthopedic hospital, we further investigated the indoor air quality in emergency rooms where air purifiers were used (AlecoAir P80 Traditio, flow rate 650 mc/h) and those that did not use these devices.

The reference range for the indoor air quality indices, including carbon dioxide, nitrogen dioxide, volatile organic compounds and particles with a diameter smaller than 2.5 μm, were defined according to the World Health Organization Guidelines [31,45,46].

In this study, we did not include human subjects, which is why we could not study the degree of exposure to pollutants of the medical staff working in the Orthopedics–Traumatology department.

The conduct of this study was approved by the Ethics Committee of the Targu Mures County Emergency Hospital (ECHTM). Gathering the data necessary for the research was carried out following the ethical principles of the Declaration of Helsinki.

### 2.2. Statistical Analysis

Statistical analysis was performed using IBM SPSS, version 29.0.2 (SPSS, Inc., Chicago, IL, USA), for Microsoft Windows. We used the recorded values of the indoor air quality indices as input data. Since there was no normal distribution, we expressed continuous variables as the median with the range. We performed the comparison of two continuous variables with the Mann–Whitney test. For the evaluation of the four continuous variables, we used the Kruskal–Wallis test. For post hoc analysis, we used Mann–Whitney test. In the case of these tests, statistical significance was defined by p being less than 0.05 (2-tailed).

## 3. Results

### 3.1. Indoor Air Quality Variations in Orthopedic Areas

**Figure 2:**
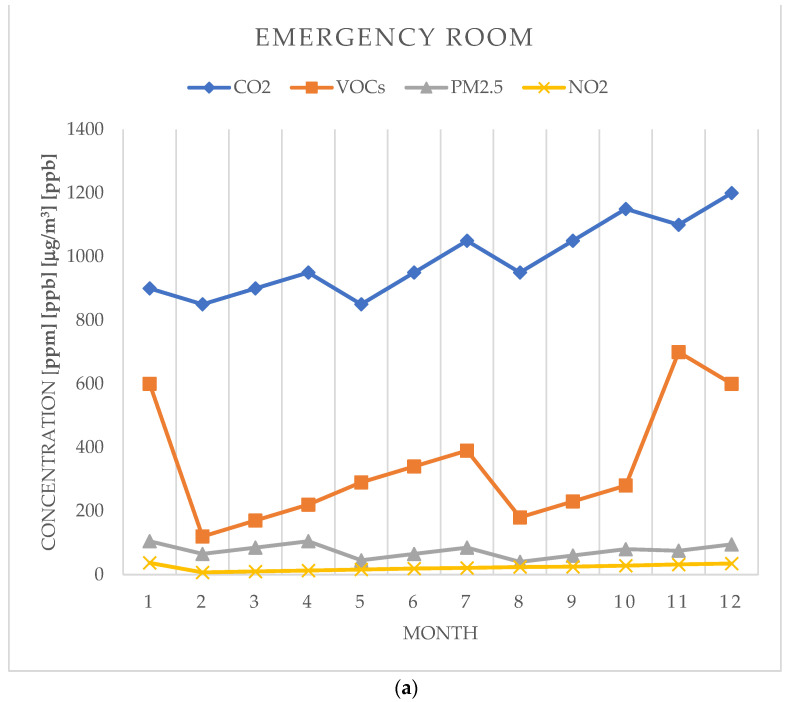

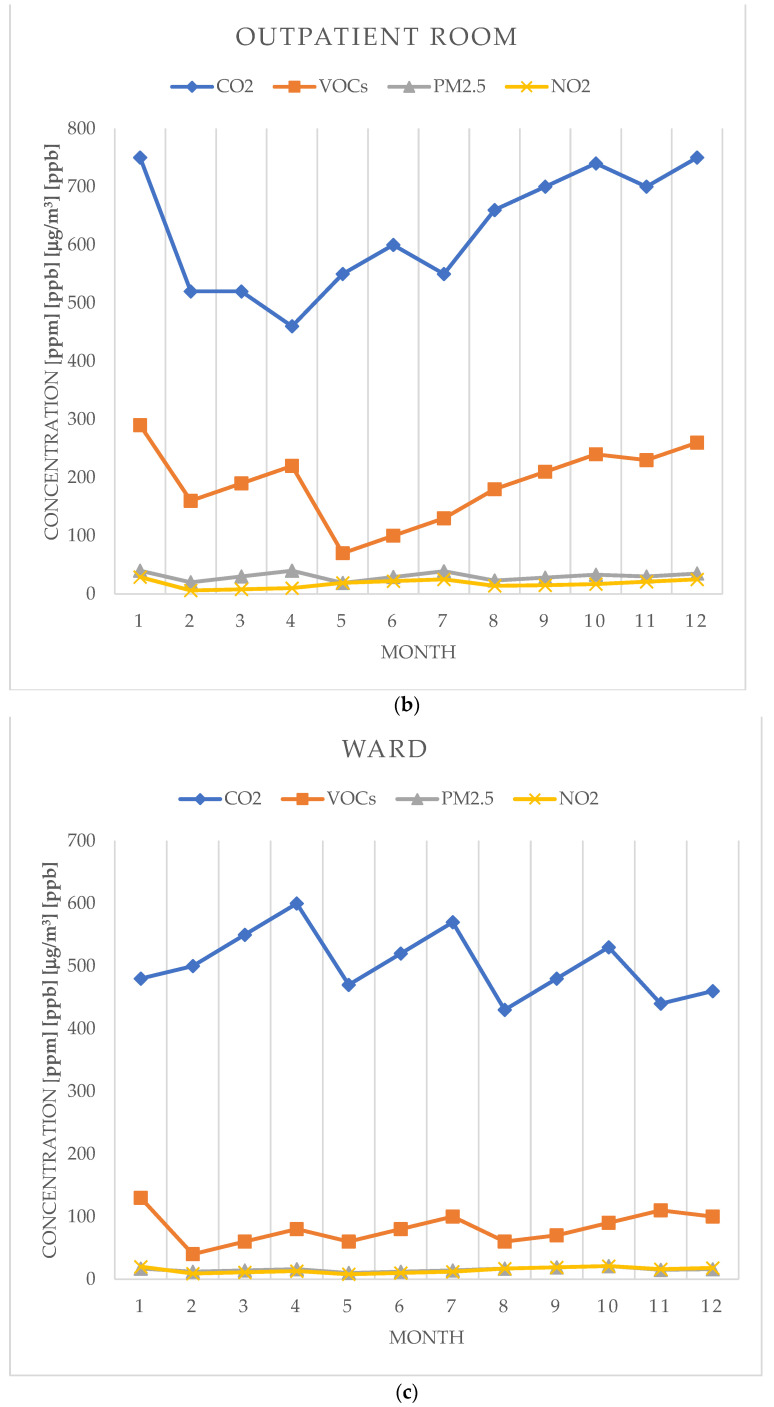
Variation in indoor air quality in Orthopedics–Traumatology wards: (**a**) outpatient room; (**b**) emergency room; (**c**) ward. Measured indoor air quality indices: CO_2_—carbon dioxide; VOCs—total volatile organic compounds; PM2.5—particles with diameter less than 2.5 μm; and NO_2_—nitrogen dioxide.

The results recorded for the measurement of the indoor air quality and the variation in the indicators in the three areas of the Orthopedics–Traumatology department are represented in Figure 2.

**Figure 3:**
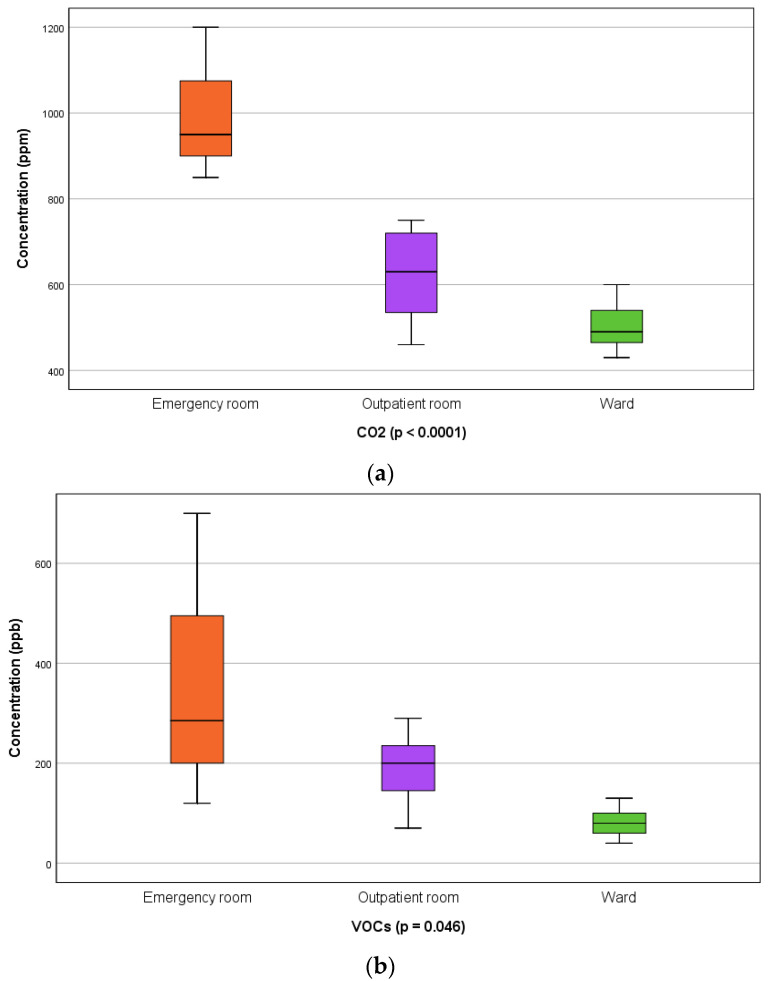

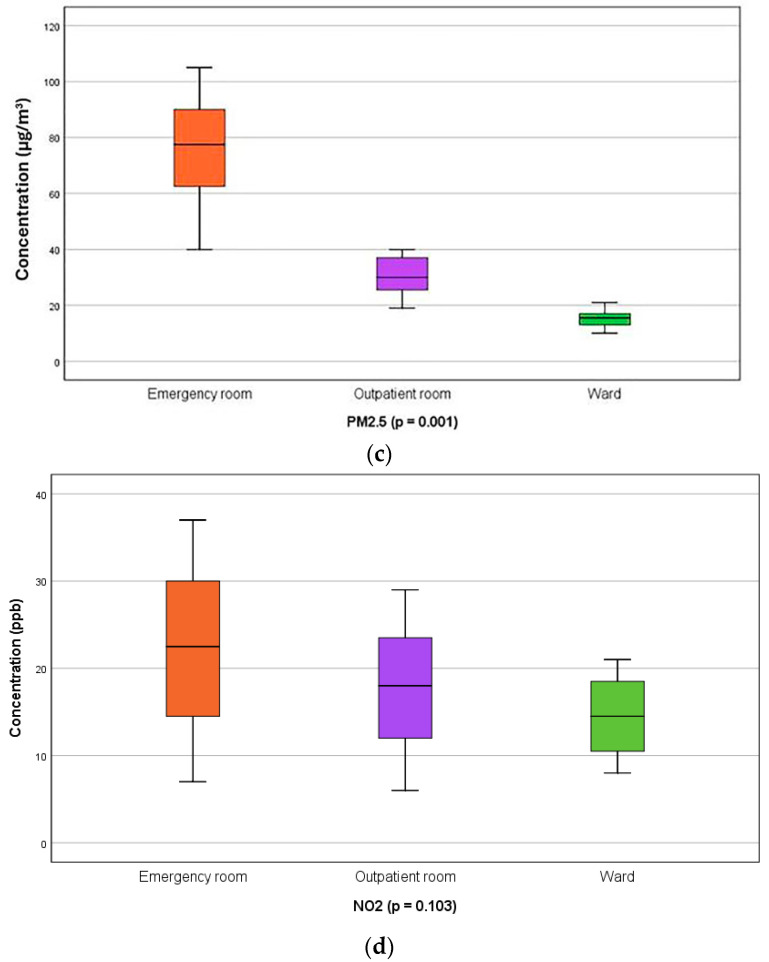
Comparison of indoor air quality concentrations between orthopedic hospital wards: (**a**) CO_2_—carbon dioxide; (**b**) VOCs—total volatile organic compounds; (**c**) PM2.5—particles with a diameter smaller than 2.5 μm; (**d**) NO_2_—nitrogen dioxide.

In the emergency room, the average annual concentration of CO_2_ was 991 ppm, and the average value of the monthly concentration was over 850 ppm. The average annual concentration in the outpatient room was 625 ppm, and that in the ward was 502 ppm (Figure 3a).

The average annual concentration of VOCs in the emergency room was 343 ppb, and this was significantly higher than in the lounge with beds where an average value of 81 ppm was recorded, as can be seen in Figure 3b. In the outpatient room, the average annual VOC concentration was 190 ppb.

In the emergency room, the average annual concentration of PM2.5 had a value of 75.4 mg/m^3^, significantly higher than that in the outpatient room, 30.5 mg/m^3^, or that in the lounge with beds, 15.25 mg/m^3^ (Figure 3c).

Regarding the annual mean NO2 concentrations, there were no significant differences between the areas analyzed, as can be seen in Figure 3d.

In Table 1, we have presented the reference ranges of the indoor air quality indices for this study according to World Health Organization Guidelines [31,45,46].

### 3.2. Seasonal Effects on Indoor Air Quality

**Figure 4:**
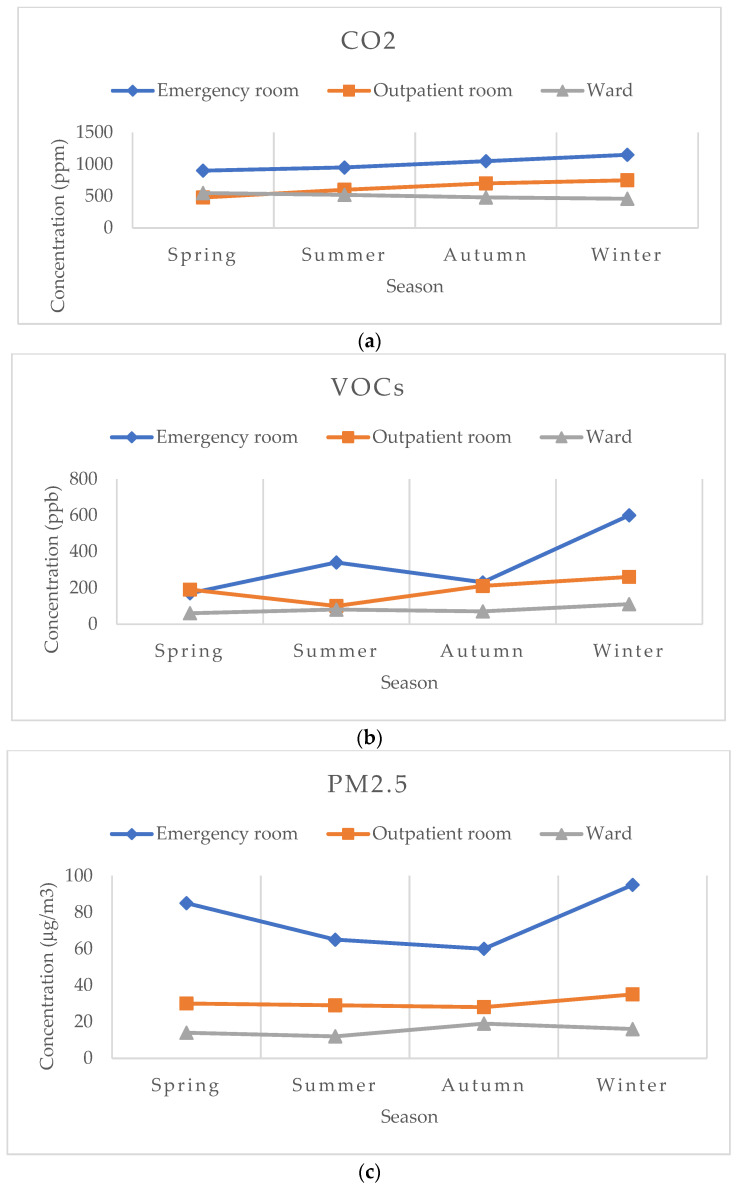

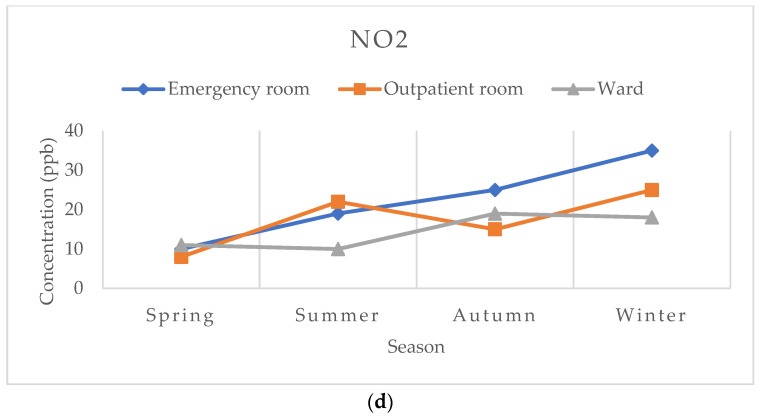
Seasonal variation in indoor air quality in different areas of the orthopedic hospital: (**a**) CO_2_—carbon dioxide; (**b**) VOCs—total volatile organic compounds; (**c**) PM2.5—particles with a diameter smaller than 2.5 μm; (**d**) NO_2_—nitrogen dioxide.

In the emergency room and outpatient room, the concentration of CO_2_ increased from spring to winter, while in the ward, it registered a slight decrease, as can be seen in Figure 4a.

In each of the three analyzed areas, the VOC concentration was higher in the winter period, compared to the other seasons (Figure 4b) (*p* = 0.0341). Moreover, we found that the concentrations of PM2.5 in the emergency room were higher in all four seasons than in the outpatient room or the ward with beds. In this area, the PM2.5 concentrations were lower in summer and autumn compared to the other seasons (Figure 4c).

As for the concentration of NO_2_, it showed seasonal variations in each of the three areas (Figure 3d). A steady increase in the NO_2_ concentration occurred from spring to winter in the emergency room. In the outpatient room, higher NO_2_ concentrations were recorded in summer and winter than in spring and autumn. On the other hand, in the ward with beds, the concentration of NO_2_ was higher in autumn and winter.

### 3.3. Effects of Using Air Filters in Indoor Air Pollution Control

**Figure 5:**
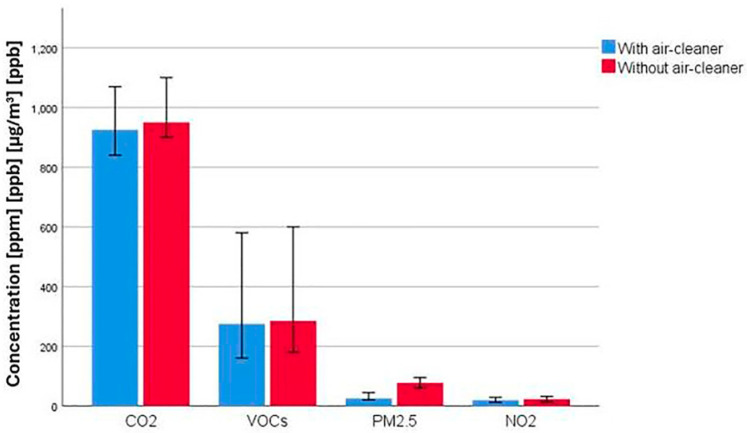
The effects of using air filters in the control of air pollution inside emergency rooms. Measured indoor air quality indices: CO_2_—carbon dioxide; VOCs—total volatile organic compounds; PM2.5—particles with diameter less than 2.5 μm; and NO_2_—nitrogen dioxide.

There were no differences between the two groups in terms of the type and number of procedures performed, as well as the average number of patients consulted. In Figure 5, we have represented the results of the measurements, which indicate a significantly lower concentration for PM 2.5 (*p* = 0.014). In the case of the other investigated pollutants, CO_2_, VOCs and NO_2_, there were no significant differences in the concentrations of the two groups (*p* = 0.13921, *p* = 0.0612 and *p* = 0.0668, respectively).

## 4. Discussion

External sources of pollution are increasingly subjected to regulated control, unlike internal sources of pollution. The latter require proper identification and control because they have the potential to worsen both indoor and outdoor air [47]. Medical staff spend a large part of their time in the public indoor environments of hospitals, and indoor air quality is an important factor that affects the health of staff as well as patients [48]. A number of procedures and activities carried out in hospitals, such as pharmaceuticals, cleaning and disinfecting compounds, sterilization procedures, biological contaminants and the use of various chemicals, affect indoor air quality in hospitals [26,49]. Our findings agree with those of the study conducted by Baurès et al. [50], which indicates that the main indoor air pollutants are CO_2_, NO_2_, VOCs, radon, sulfur dioxide, ozone, PM, toxic metals and microorganisms. This study aimed to evaluate the indoor air quality in the wards of an orthopedic hospital, as an integral part of an emergency hospital with exposure to several indoor pollutants.

In our study, we found that the emergency room had significantly higher concentrations of CO_2_, VOCs and PM2.5 compared to the outpatient room or ward. We also detected seasonal differences in the indoor air quality indicators, which indicated a continuous increase in the CO_2_ concentration from spring to autumn in the emergency room and outpatient room. Also, the VOC concentration was higher in the winter period, compared to the other seasons, in all three hospital wards. The PM2.5 concentrations in the emergency room were lower in summer and autumn compared to other periods of the year. Also in the emergency room, from spring to winter, the concentration of NO_2_ had a constant increase, unlike the ward, where this concentration was higher in autumn and winter. Installing an air purifier in the emergency rooms, which were the most polluted orthopedic areas, had significant effects only on the PM2.5 concentrations, without any effects on the CO_2_, VOC and NO_2_ concentrations. Therefore, it is advisable to install ventilation systems in emergency rooms that, by maintaining the quality of the indoor air, create a safe and healthy environment. At the same time, measures must be taken to ensure an adequate rate of air exchange in the rooms and the use of air filters intended for hospitals, as well as the regular monitoring and maintenance of the air filtration system.

CO_2_ is a major human metabolite and a constituent of the Earth’s atmosphere with an estimated concentration of 400 ppm [51]. To prevent any risk of illness, some standardized recommendations indicate concentrations of less than 700 ppm for indoors [11]. However, the most common limit indicated by most studies is 1000 ppm [52]. Exposure to values greater than 1000 ppm of CO_2_ concentration affects cognitive performance in terms of decision-making and problem-solving but also children’s respiratory performance [53]. In our study, the average CO_2_ concentration in the emergency room was higher than 850 ppm every month and higher than the other areas investigated. Therefore, due to the increased concentration of CO_2_, medical personnel working in the emergency room are exposed to greater risks of health consequences.

Volatile organic compounds (VOCs) contain a variety of chemicals, some of which are toxic, like formaldehyde, toluene, benzene, xylenes and ethylbenzene [54]. Of these, formaldehyde is the most prevalent, at 68%, and has the highest cancer risk [55]. Indoors, VOCs are generated by building materials, indoor chemical reactions, outdoor air supply, and also human activities such as cooking, smoking, the use of cleaning products and personal care [56]. Epidemiological investigations have reported a higher likelihood of cancer due to certain VOCs, such as formaldehyde, benzene and polycyclic aromatic hydrocarbons [57]. Our study highlighted higher average VOC concentrations in the emergency room, compared to the other wards analyzed. Also, the VOC concentrations increased during the winter period in all analyzed areas. The higher levels of VOCs in the emergency room can be explained by the frequent cleaning and disinfection of the premises with various disinfectants and chemical detergents but also the dust emissions from plaster that persist in the absence of proper ventilation.

Particulate matter (PM) is classified into PM2.5 and PM10, as the sizes of the particles that compose them are below 2.5 μm and below 10 μm, respectively. They are made up of a mixture of tiny particles and liquid droplets [58]. Exposure to PM2.5 is linked to many negative consequences that affect the immune system and expose people to respiratory disease, cardiovascular disease, insulin resistance, systemic inflammation and neurotoxic consequences. PM accumulates indoors due to the performance of some activities but also through migration from the outside environment [59]. In our study, we found that in the emergency room the PM2.5 concentrations were significantly higher than in the other analyzed areas. The values were also higher in spring and winter. We explain this result by plastering procedures that require the application of gypsum prepared from powder and that can contribute to high concentrations of PM2.5 in the emergency room. We explain the seasonal differences by the higher number of patients who require cast immobilization in the spring and winter seasons, with the increase in the frequency of fractures mainly due to meteorological factors in these seasons. In agreement with the study conducted by Chen et al. [60] we also found a decrease in the PM2.5 concentration when using air purifiers.

NO_2_ is considered a primary pollutant, which along with nitrogen oxide (NO) comes from combustion sources such as heaters and stoves. But in the atmosphere, NO oxidizes instantly and forms NO_2_ [61]. Exposure to NO_2_ can cause bronchopneumonia or bronchitis, lead to decreased lung function and cause a risk of Parkinson’s disease [62,63]. In our study, we found no significant differences in the NO_2_ concentrations in the orthopedic hospital areas. However, in the emergency room and the ward, the total concentration of NO_2_ was 80% higher during the winter compared to the summer. We explain this finding by the continuous use of heaters during the winter period.

Our study has some limitations. First, we did not involve human subjects to study the health effects of medical personnel’s exposure to air pollutants. Due to this limitation, we could not provide concrete evidence regarding the direct relationship between indoor air quality and the health of medical staff or patients in the orthopedic hospital. Another limitation stems from the fact that this study was conducted in a single emergency hospital that performs certain types of orthopedic procedures and uses materials obtained from a limited number of providers. For this reason, it is a pilot study whose strength is the additional evidence it provides regarding the need to improve indoor air quality in orthopedic hospitals, and especially in emergency rooms, to protect the health of medical staff and patients. Findings may vary in other hospitals that perform other orthopedic medical procedures such as fiberglass casts and have other sources of supply. Considering these identified limitations, we propose to continue the study in the future by including in the research other hospitals at the regional or national level which have different types of orthopedic medical procedures in their portfolio, and which have supplies of medical materials from various suppliers. In this way, a study can be carried out at the national level in different periods of time, which could therefore have different conclusions. Through this, indoor pollution trends in orthopedic hospitals could be tracked and policies could be formulated so that medical institutions and their staff are increasingly oriented towards ecological sustainability. Also, we have investigated primary target pollutants in hospitals, namely CO_2_, NO_2_, VOCs and PM2.5, but further studies may investigate other pollutants as well, like nitrous oxide, carbon oxide particles with a diameter of 10 μm or less, etc. Other inaccuracies of this study may appear due to the level of external pollution that could influence the measurements inside the hospital. To increase the accuracy and results of research in the future, it would be desirable to explore the influences of the external environment, which can also be evaluated as the hospital is in the commercial, residential, and industrial belts of the city.

## 5. Conclusions

This study monitored indoor air quality in an orthopedic emergency hospital by comparing indoor air quality indices in its areas. The results of the study indicate significantly higher CO_2_, VOC and PM2.5 concentrations in the emergency room compared to the outpatient room or ward. We also found seasonal differences in the indoor air quality indices. In the emergency room and outpatient room, the concentration of CO_2_ increased from spring to winter. In the emergency room, the concentrations of PM2.5 were lower in summer and autumn compared to the other seasons, but in each season, they were much higher than in the outpatient room or the ward. The VOC concentration in each area was higher in the winter period compared to the other seasons. The concentration of NO_2_ in the emergency room increased from spring to winter.

Improving the indoor air quality in the hospital can be achieved by measures to reduce the consumption of substances that directly affect it, or by replacing them with more environmentally friendly ones, for cleaning and disinfecting instruments; cleaning wounds with solutions, antiseptics and sterile dressings; local anesthesia; medication administration; etc. The use of various chemicals, the use of cleaning products and the frequent cleaning and disinfection of the premises with various disinfectants and chemical detergents must be carefully analyzed. The use of heaters, fans, coolers or additional personal cleaning devices should also be avoided. An important role in maintaining air quality is played by ventilation systems in hospitals that facilitate the maintenance of a safe and healthy environment. This requires the use of air filters designed for hospitals, the regular maintenance and monitoring of the air filtration system and the adoption of measures to ensure that there is an adequate rate of air exchange in the hospital.

The findings of this study indicate that orthopedic hospitals and especially emergency rooms are exposed to the risk of indoor air pollution. As this situation could affect the health of medical staff and patients, decision makers should consider orthopedic medical practice guidelines and aspects related to indoor air pollution in applying appropriate protective measures to neutralize the effects of poor indoor air quality. The improvement of working conditions for specialist doctors, first, requires the periodic evaluation of their occupational disease risks; it also requires the application of solutions to reduce the risks by limiting the degree of exposure to chemical substances for treatment and disinfection, restricting the duration of time spent in the emergency room, etc.

## Figures and Tables

**Table 1:** Indoor air quality indices detailed reference range.

Indoor air Quality Indices Reference Range	CO_2_—Carbon Dioxide [ppm]	VOCs—Volatile Organic Compounds [ppb]	PM2.5—Particles with a Diameter Smaller Than 2.5 μm [μg/m^3^]	NO_2_—Nitrogen Dioxide [ppb]
Poor quality value range	>1500	>800	>100	>250
Tolerable value range	800–1500	400–800	50–100	100–250
Acceptable value range	400–800	0–400	<50	<100

## Data Availability

The data used in this study can be requested from the corresponding author.
